# What Is Stopping the Use of Genetically Modified Insects for Disease Control?

**DOI:** 10.1371/journal.ppat.1005830

**Published:** 2016-10-06

**Authors:** Anusha Panjwani, Anthony Wilson

**Affiliations:** The Pirbright Institute, Pirbright, Woking, Surrey, United Kingdom; University of Pittsburgh, UNITED STATES

Insect-borne pathogens impose a substantial burden on health, the environment, and agricultural production, and rapid outbreaks of such pathogens are becoming more common [e.g., 1,2]. Population control is an important component of strategies to control insect-borne pathogens. However, some technologies such as insecticide use are becoming less effective due to resistance [3], or their use is increasingly restricted due to environmental legislation [e.g., 4].

An emerging technology that could form part of strategies for controlling insect-borne pathogens is to suppress or replace insect populations by releasing genetically modified (GM) insects. Trial releases of GM insects for disease control have now been conducted in the Caribbean [5], Malaysia [6], and Brazil [7], but none have yet proceeded in the European Union (EU). In 2015, the only active European GM insect application (olive fly) was withdrawn two years after submission in response to the cost of additional experiments requested by the regulators [8]. In the same year, the regulatory bodies of Brazil and the United States approved two products that were based on a similar mechanism [9,10]. The EU assessment process has been criticised as slow, expensive, unpredictable, and as deterring innovation and technology transfer in this area [11–13]. A recent United Kingdom parliamentary inquiry into the subject concluded that, although appropriate risk assessment was essential, current EU implementation was preventing this technology from realising its full potential [14].

One reason behind geographical differences in the assessment process is probably that there are no truly global guidelines. The Cartagena Protocol [15] establishes basic principles for assessing the safety of genetically modified organisms (GMOs) intended for deliberate release, but some countries with significant involvement in this area such as the US and Argentina are not signatories, and the framework for testing GM mosquitoes published by the World Health Organization (WHO) is explicitly not intended as guidance [16]. There are therefore significant regional differences in interpretation and implementation. For example, assessment in the EU considers only risks [17,18], whereas in the US, Brazil, and New Zealand, potential benefits are also considered [19,20]. Another difference is whether assessment considers the process by which the product is created. Identical products created by classical mutagenesis or GM would be assessed differently in the EU [21,22] but not Canada [23,24].

Regional differences also reflect public opinion. The history of field releases of sterilised disease vectors and GMOs illustrates the importance of effective dialogue with the public. In the 1970s, public concerns about releases conducted by the WHO Research Unit on Genetic Control of Mosquitoes in New Delhi were a major factor in the project’s cancellation [25]. More recently, there have been high-profile anti-GM protests in the UK and Germany [26]. Elsewhere, pro-GM attitudes in Pakistan led to widespread smuggling and planting of GM cotton before its official approval in 2010 [27].

Part of the solution could be providing more information to the public. Low public awareness of the New Delhi project is thought to have contributed to opposition [25], and the Cartagena Protocol requires that signatories facilitate public awareness of GMOs and their use [15]. However, despite efforts to inform the local community before trialling GM mosquitoes in Grand Cayman in 2009 [28], there were subsequently complaints that the international research community was not adequately consulted [28,29], highlighting the importance of engaging with all stakeholders and not just beneficiaries. More recently, attempts to inform the public about GM mosquito releases in Brazil did not prevent the spread of conspiracy theories linking them to the emergence of Zika virus [30–32].

It is also important that communication is two-way. The “knowledge deficit” model of science communication is increasingly recognised as outdated [33], partly because public concerns are not always obvious. In the UK, discussion at recent public and parliamentary events on the use of GM insects for disease control organised by the authors [34,35] suggested that concerns were higher where private companies were involved than for public sector research. Elsewhere in Europe, it has been suggested that, although public debate around GMOs often focuses on possible biological risks, actual concerns may be about the economic consequences of their use [26]. If true, this could create a vicious cycle: public mistrust of GM driven by its concentration in the hands of a few companies could encourage policymakers to make the licensing and approval process more restrictive, driving up regulatory costs and excluding small companies [14]. However, the views of the most vocal opponents of GM do not necessarily represent views held at a population level [36,37].

Given the lessons learned so far, we suggest that the following three areas represent the best compromise between proportionate risk assessment and maximising the potential benefits of GM.

Firstly, assessments should regulate product, not process. Currently, the EU regulates by process, meaning that mosquitoes rendered sterile by radiation, genetic modification, or the addition of symbiotic bacteria such as *Wolbachia* [38,39] would be regulated differently and potentially by a different agency. Product-based regulation works effectively in countries such as Canada.

Secondly, assessments should consider the balance of risk and benefit resulting from GM insect release, not risk alone. Currently, the EU only considers risk (as discussed above), preventing applicants from providing information on the benefits of the proposed technology [40]. Risk assessment should consider the risks of inaction (status quo) as well as those of proposed action and realistic alternatives such as insecticides [41].

Finally, the process of assessment needs to be more transparent. This would allow both beneficiaries and stakeholders to take part in better-informed discussions of proposed products and the results of trial studies, encourage consistent assessment by authorities across multiple regions, and make it clear to the public that assessments receive expert scrutiny, building confidence in the process. At the same time, we recognise that requiring full disclosure of methods and data at the time of application would strongly discourage innovation. Possible compromises could be to only release data from successful assessments, to embargo these data for a period after the assessment is approved, and/or to consider some form of market exclusivity for a period in return for such disclosure.

Issues relating to the optimal balance between risk and benefit for the use of GM insects for disease control are not going away. New technologies are making it easier and faster to develop GM insects [42]. Although appropriate risk assessment is necessary for novel technologies, focusing assessment on poorly defined biological risks for which there may be no plausible mechanism discourages small companies and academic organisations from developing transgenic insect technologies. The suggestions above could accelerate risk assessment without increasing risk and facilitate more intelligent dialogue between proponents and opponents of the technology.
